# Supplementary material for: The Burden of Post-Translational Modification (PTM)—Disrupting Mutations in the Tumor Matrisome
Source: Cancers (Basel). 2021 Mar 3;13(5):1081. doi: 10.3390/cancers13051081 (PMC7959462; doi:10.3390/cancers13051081)
Supplement: Supplementary file 1 [file cancers-13-01081-s001.zip › cancers-1117378-supplementary_VI.docx]

Article

The Burden of Post-Translational Modification (PTM)—
Disrupting Mutations in the Tumor Matrisome

Elisa Holstein ^1,†^, Annalena Dittmann ^1,†^, Anni Kääriäinen ^1^, Vilma Pesola ^1^, Jarkko Koivunen ^1^, Taina Pihlajaniemi ^1^, Alexandra Naba ^2,3^ and Valerio Izzi ^1,4,5,^*

| **Citation:** Holstein, E.; Dittmann, A.; Kääriäinen, A.; Pesola, V.; Koivunen, J.; Pihlajaniemi, T.; Naba, A.; Izzi, V. The Burden of Post-Translational Modification (PTM)—Disrupting Mutations in the Tumor Matrisome. *Cancers* **2021**, *13*, x. https://doi.org/10.3390/xxxxx  Academic Editors: Nikos Karamanos and Zoi Piperigkou  Received: date  Accepted: date  Published: date  **Publisher’s Note:** MDPI stays neutral with regard to jurisdictional claims in published maps and institutional affiliations.  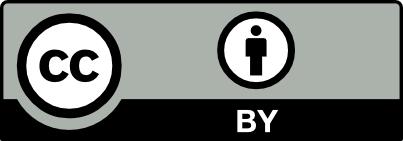  **Copyright:** © 2021 by the authors. Submitted for possible open access publication under the terms and conditions of the Creative Commons Attribution (CC BY) license (http://creativecommons.org/licenses/by/4.0/). |
| --- |

^1^ Faculty of Biochemistry and Molecular Medicine, University of Oulu, FI-90014 Oulu, Finland;
elisa.holstein@gmx.at (E.H.); annalena.dittmann@oulu.fi (A.D.); anni.kaariainen@oulu.fi (A.K.);
vilma.pesola@oulu.fi (V.P.) jarkko.koivunen@oulu.fi (J.K.); taina.pihlajaniemi@oulu.fi (T.P.)

^2^ Department of Physiology and Biophysics, University of Illinois at Chicago, Chicago, IL 60612, USA; anaba@uic.edu

^3^ University of Illinois Cancer Center, Chicago, IL 60612, USA

^4^ Faculty of Medicine, University of Oulu, FI-90014 Oulu, Finland

^5^ Finnish Cancer Institute, 00130 Helsinki, Finland

***** Correspondence: valerio.izzi@oulu.fi

† These authors contributed equally to this work.


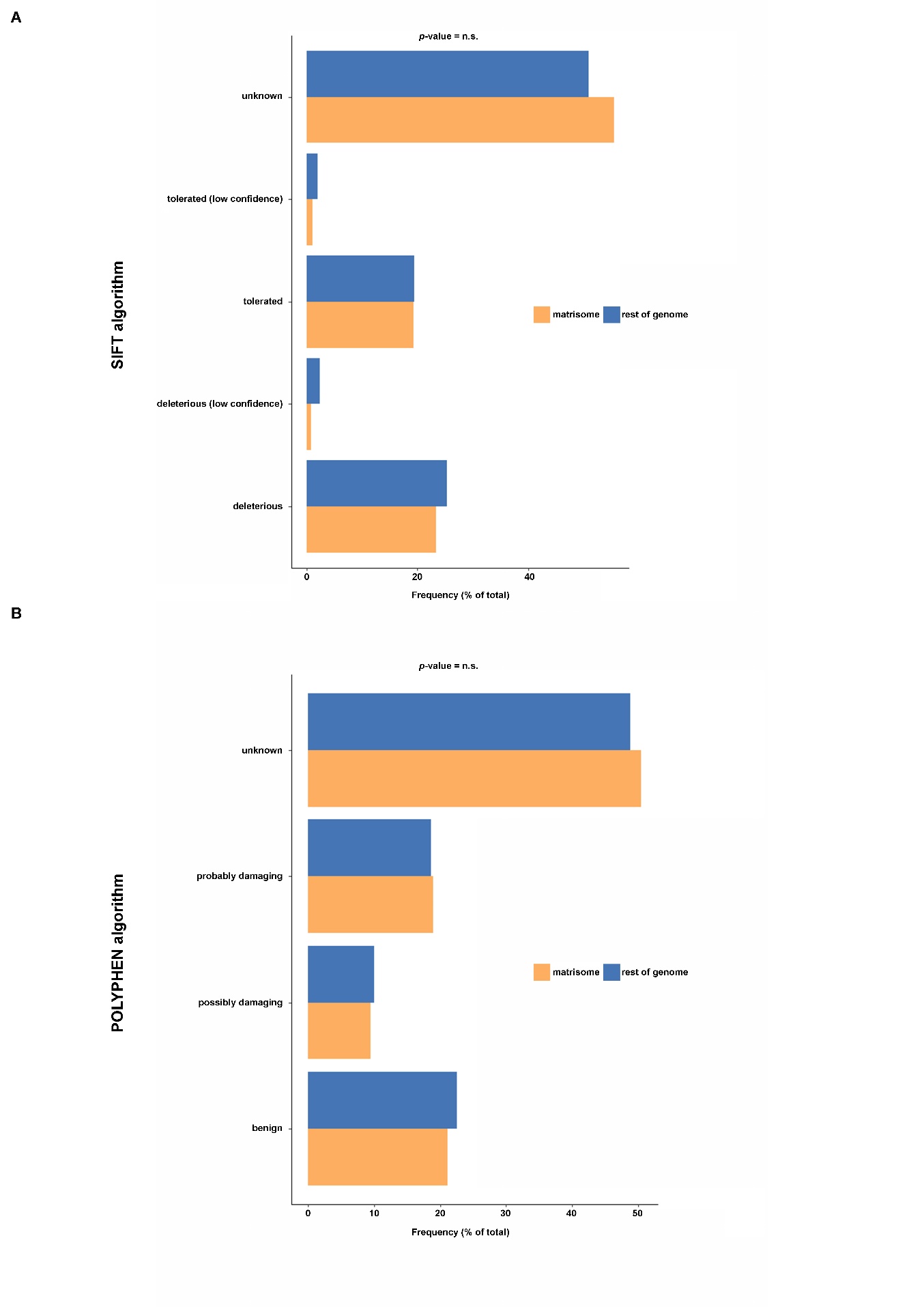


**Figure S1.** Effect of mutations in the tumor matrisome vs*.* rest of the genome. The effects of all mutations on the produced proteins were modelled according to (**A**) SIFT and (**B**) POLYPHEN algorithm and compared between matrisome and non-matrisome genes. Abbreviations: n.s., not significant. *p*-values are from Chi-square tests.


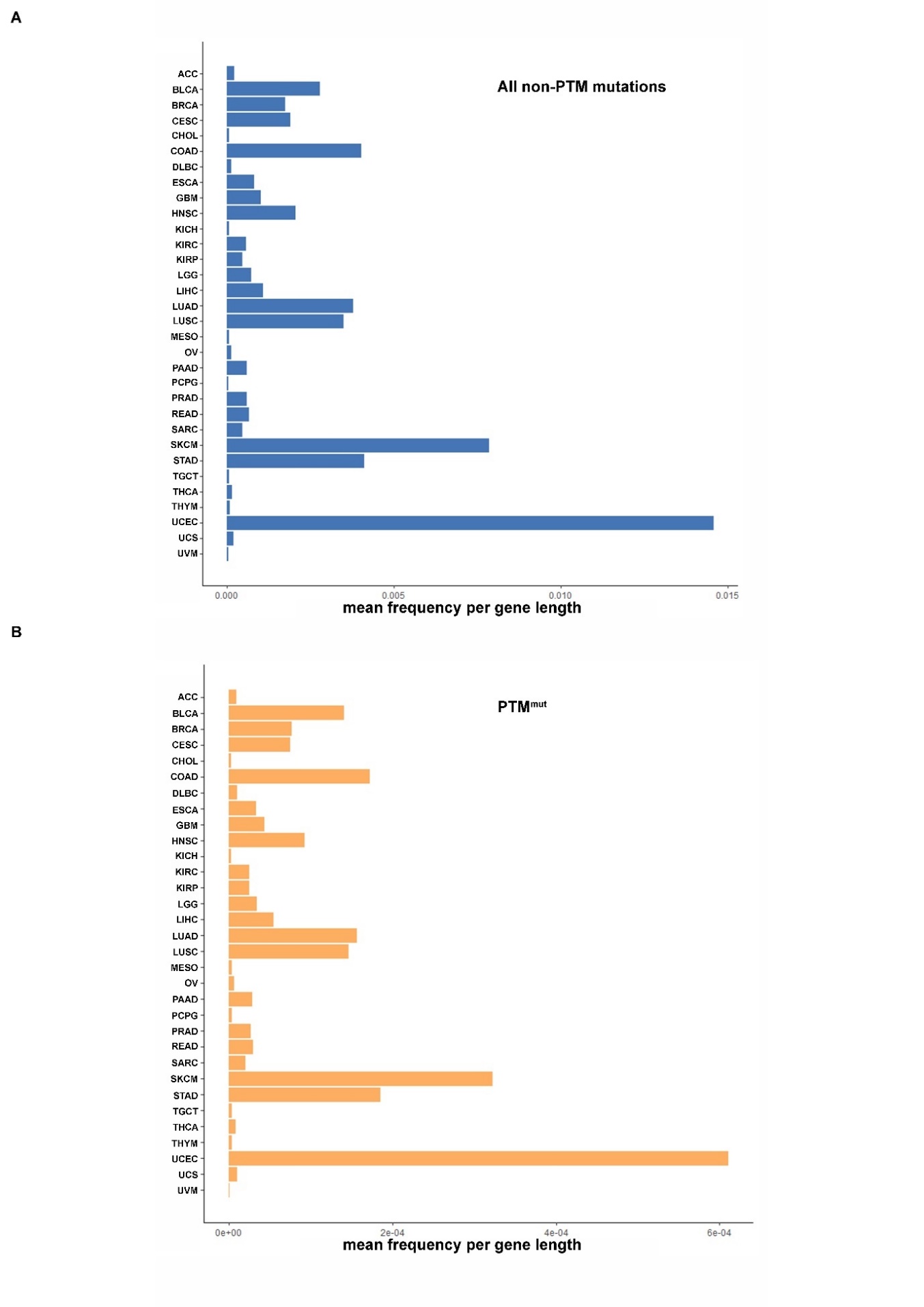


**Figure S2.** Frequency of PTM^mut^ normalized by length of gene. The ratio of mutations not affecting PTM sites (non-PTM) by gene length was calculated across tumor types (**A**) and compared with the same ratio for mutations affecting PTM sites (**B**). Notice the close similarity between the two ratios tumor-wise.


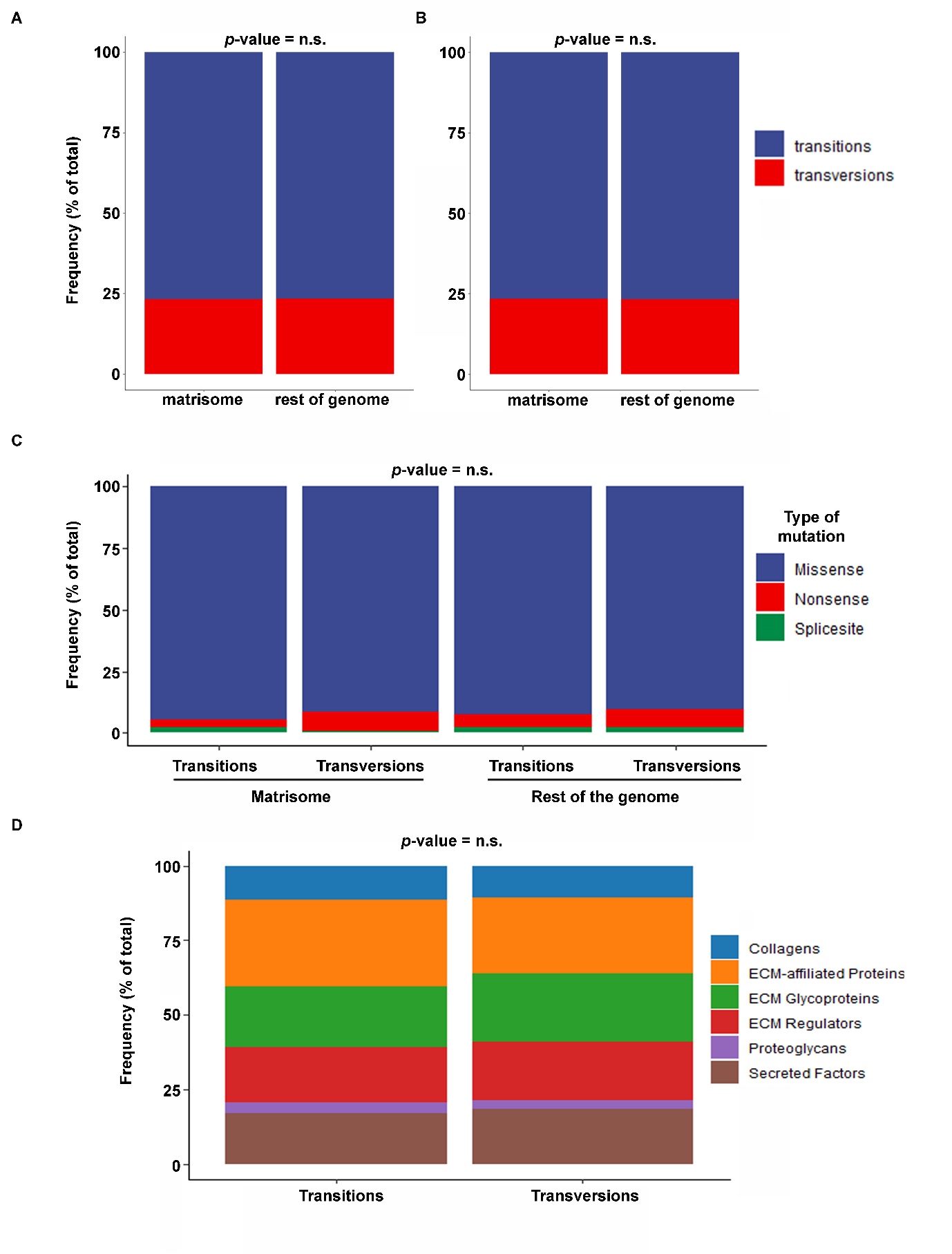


**Figure S3.** Transitions and transversions among PTM^mut^ in the tumor matrisome. The % amount of transitions and transversions in PTM^mut^ (**A**) and (**B**) in non-PTM mutations was calculated and compared for matrisome and rest of the genome. The same values were further tabulated across different types of mutation (**C**) and, (**D**), transitions and transversions within PTM^mut^ of the matrisome were compared across matrisome families. Abbreviations: n.s., not significant. *p*-values are from Chi-square tests.


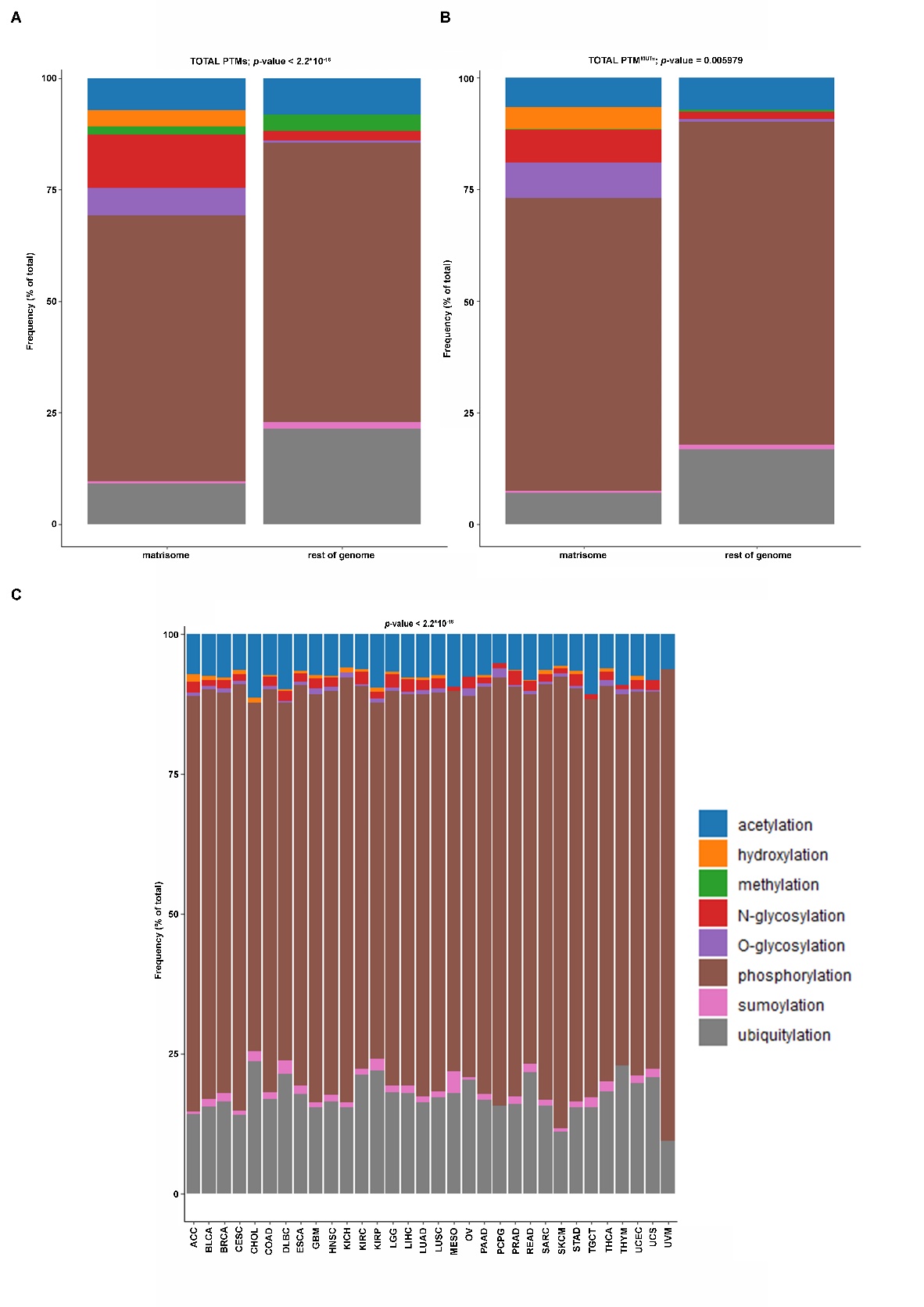


**Figure S4.** Development and calculation of the PTM^mut^ “burden”. The amount of mutations across loci with different types of PTM in the matrisome and rest of the genome (**A**) and the different baseline quantities of such PTMs in the two groups (**B**) were used to calculate a PTM-specific ratio (the “burden”) across the different tumor types (“local burdens”, only matrisome shown in **C**). *p*-values are from Chi-square tests.
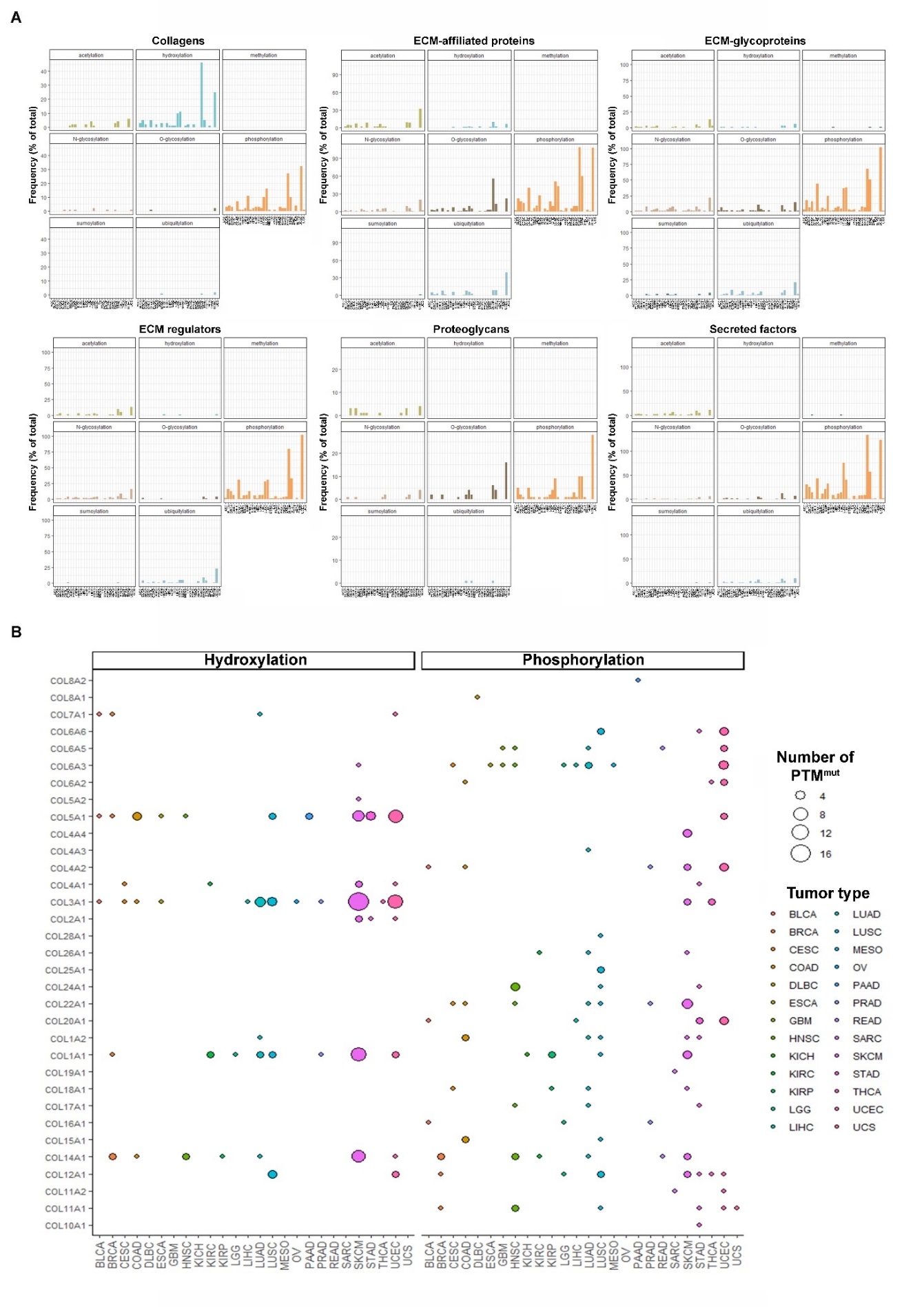
.

**Figure S5.** Variety and abundance of PTM^mut^ in the tumor matrisome**.** The amount of PTM^mut^ across the different matrisome categories and tumor types (**A**) varies considerably (*p*-value < 2e-16 for all, Chi-square test) though similar patterns of PTM^mut^ acquisition in genes can be found across multiple cancers, depending on the type of PTM (**B**).

**
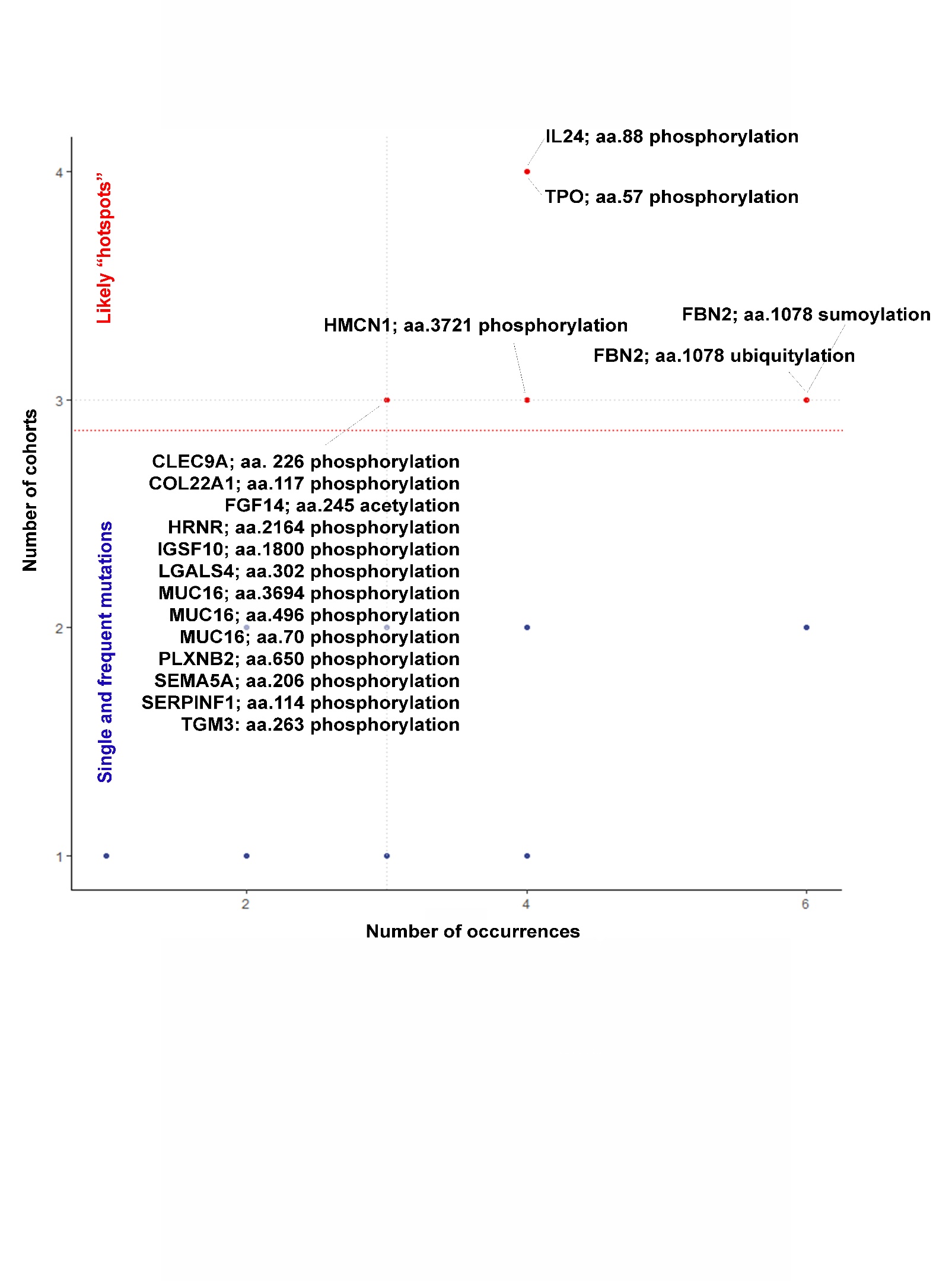
 Figure S6.** PTM^mut^ “hotspots” in the tumor matrisome. The occurrence of each matrisome PTM^mut^ for the total Pan-Cancer cohort (number of occurrences) and the number of cohorts in which it appeared (number of cohorts) were calculated, and the likely “hotspots” (those occurring at least once in three cohorts or more) were identified (in red).


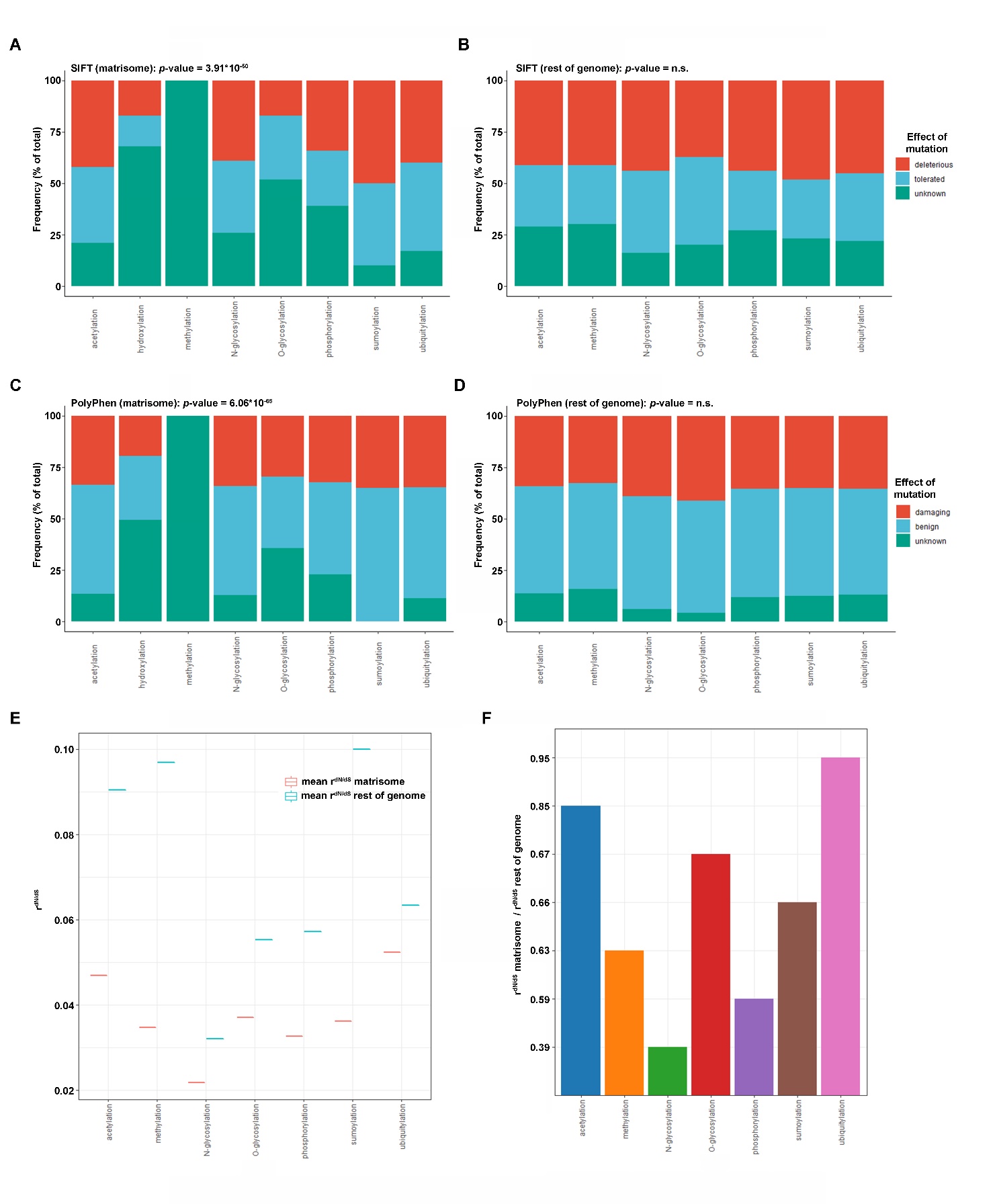


**Figure S7.** Mutational effects and conservation of PTM^mut^ in the tumor matrisome. The effects of mutations across loci with different types of PTM were calculated for the tumor matrisome and rest of the genome using SIFT and POLYPHEN algorithms (**A**–**D**). Note the lower abundance of deleterious or damaging PTM^mut^ in the tumor matrisome. In (**E**), the ratio of PTM^mut^ to silent mutations for each gene (rdN/dS) was calculated and then averaged for the Pan-Cancer cohort across the different types of mutations for the matrisome and rest of the genome, and (**F**) compared (matrisome vs. rest of the genome) to evaluate differences. Abbreviations: n.s., not significant. *p*-values are from Chi-square tests.

**
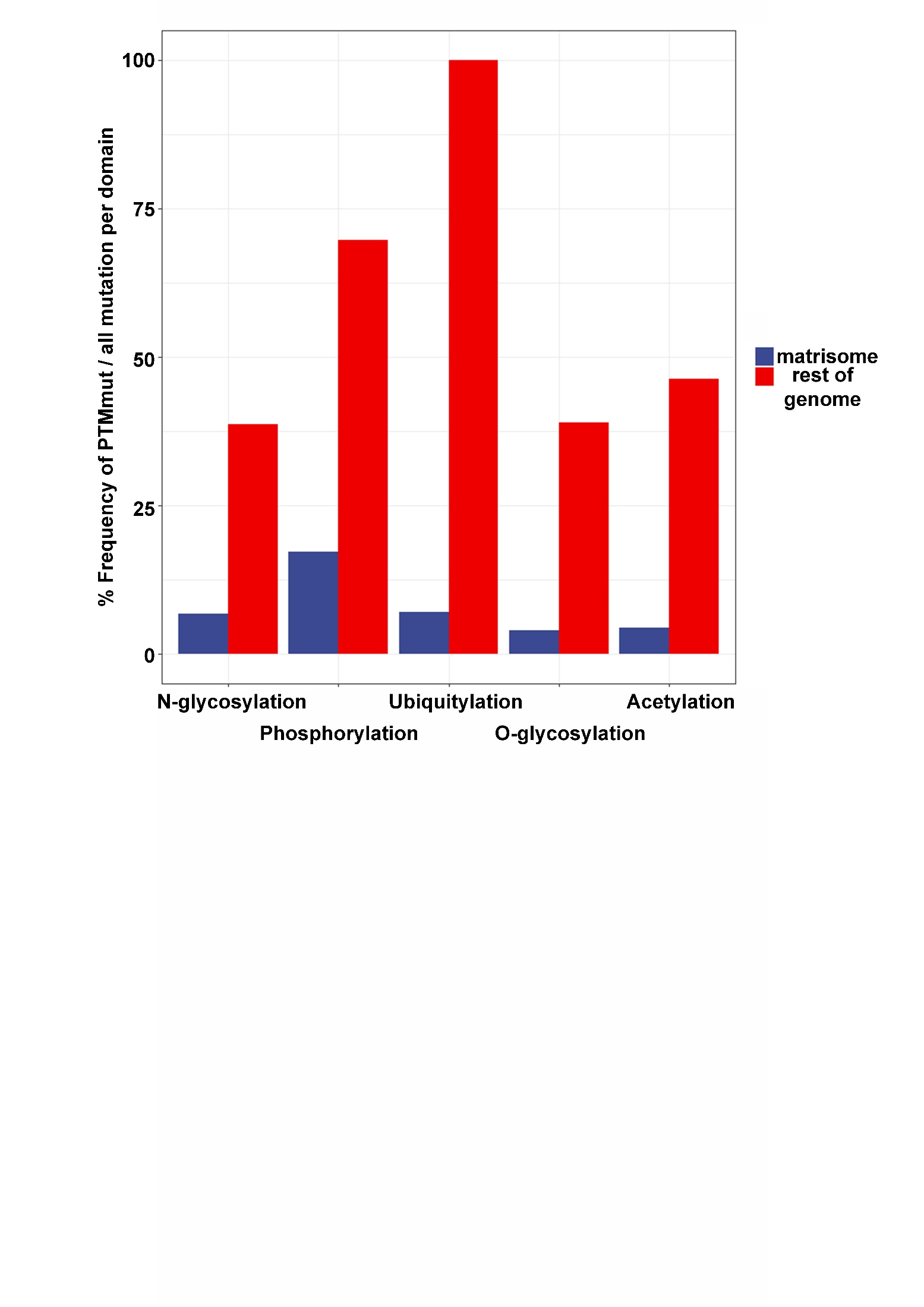
Figure S8.** Mapping and abundance of PTMmut at the protein domain level. Each PTMmut and non-PTM mutation in the tumor matrisome or in the rest of the genome was mapped to protein domains according to Pfam coordinates and the % of mapping mutations (PTMmut vs. all mutations) was calculated to compare the relative tolerance of any protein domain to PTMmut across different types of PTM in the two groups.


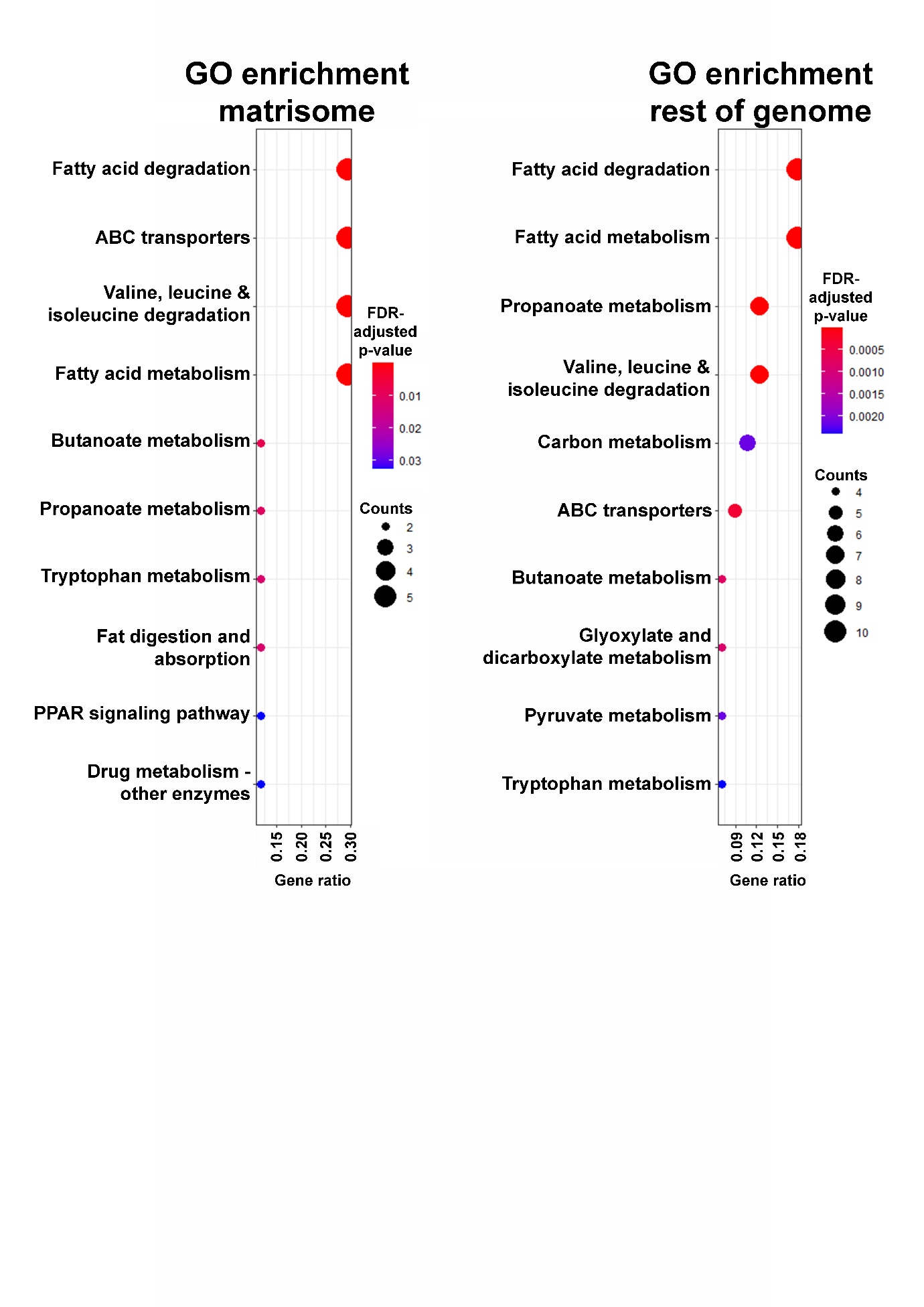


**Figure S9.** Ontological enrichment of PTM^mut^-abundant protein domains. Protein domains (from Pfam) whose PTM^mut^ vs. all mutations ratio was at least 2 times higher in (left) the tumor matrisome than in the rest of the genome or, (right) vice versa, were mapped back into the genes of origin and these subjected to ontological enrichment using the Kyoto Encyclopedia of Genes and Genomes (KEGG) annotations. Only terms with false discovery rate (FDR) < 0.01 were maintained.


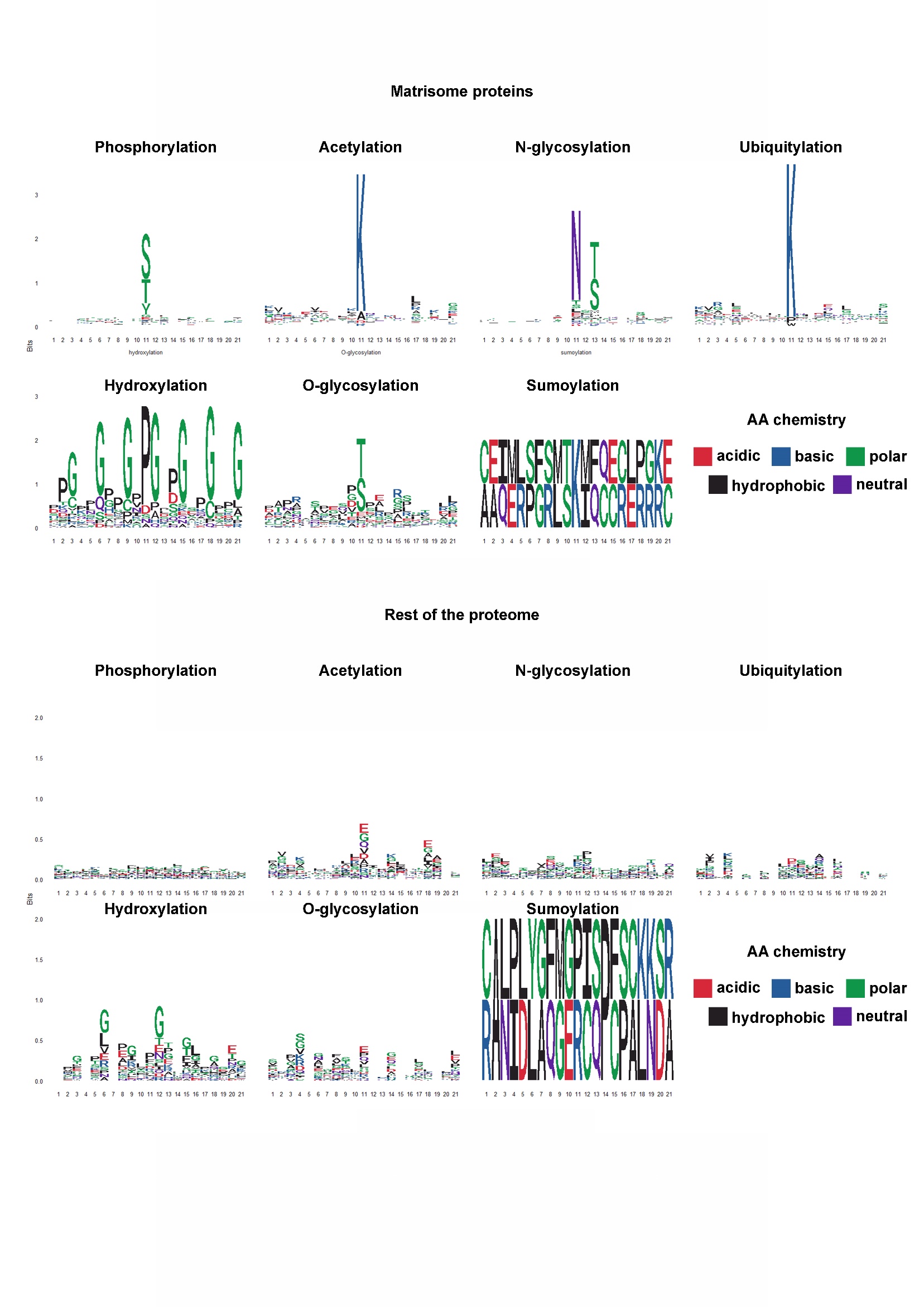


**Figure S10.** Sequence features of PTM-mutated loci. Amino acid (AA) positions affected by PTM^mut^ in the matrisome and rest of the genome were expanded by 10 AA in both directions and consensus sequences (logos) were generated according to the relative frequency of AA in each position and their chemistry. Note that, due to the different length of the protein isoforms the mutations map to, the AA affected by PTM^mut^ is always in position 11 or 12.
